# Dusp3 deletion in mice promotes experimental lung tumour metastasis in a macrophage dependent manner

**DOI:** 10.1371/journal.pone.0185786

**Published:** 2017-10-11

**Authors:** Maud Vandereyken, Sophie Jacques, Eva Van Overmeire, Mathieu Amand, Natacha Rocks, Céline Delierneux, Pratibha Singh, Maneesh Singh, Camille Ghuysen, Caroline Wathieu, Tinatin Zurashvili, Nor Eddine Sounni, Michel Moutschen, Christine Gilles, Cécile Oury, Didier Cataldo, Jo A. Van Ginderachter, Souad Rahmouni

**Affiliations:** 1 Immunology and Infectious Disease Unit, GIGA-I3, University of Liège, Liège, Belgium; 2 Laboratory of Cellular and Molecular Immunology, Vrije Universiteit Brussel, Brussels, Belgium; 3 Laboratory of Myeloid Cell Immunology, VIB inflammation research center, Ghent, Belgium; 4 Laboratory of Tumour and Developmental Biology, GIGA-Cancer, University of Liège, Liège, Belgium; 5 Laboratory of Thrombosis and Haemostasis, GIGA-Cardiovascular Sciences Unit, University of Liège, Liège, Belgium; Beijing Cancer Hospital, CHINA

## Abstract

*Vaccinia*-H1 Related (VHR) dual-specificity phosphatase, or DUSP3, plays an important role in cell cycle regulation and its expression is altered in several human cancers. In mouse model, DUSP3 deletion prevents neo-angiogenesis and b-FGF-induced microvessel outgrowth. Considering the importance of angiogenesis in metastasis formation, our study aimed to investigate the role of DUSP3 in tumour cell dissemination. Using a Lewis Lung carcinoma (LLC) experimental metastasis model, we observed that DUSP3^-/-^ mice developed larger lung metastases than littermate controls. DUSP3^-/-^ bone marrow transfer to lethally irradiated DUSP3^+/+^ mice was sufficient to transfer the phenotype to DUSP3^+/+^ mice, indicating that hematopoietic cells compartment was involved in the increased tumour cell dissemination to lung tissues. Interestingly, we found a higher percentage of tumour-promoting Ly6C^int^ macrophages in DUSP3^-/-^ LLC-bearing lung homogenates that was at least partially due to a better recruitment of these cells. This was confirmed by 1) the presence of higher number of the Ly6B^hi^ macrophages in DUSP3^-/-^ lung homogenates and by 2) the better migration of DUSP3^-/-^ bone marrow sorted monocytes, peritoneal macrophages and bone marrow derived macrophages (BMDMs), compared to DUSP3^+/+^ monocytes, macrophages and BMDMs, in response to LLC-conditioned medium. Our study demonstrates that DUSP3 phosphatase plays a key role in metastatic growth through a mechanism involving the recruitment of macrophages towards LLC-bearing lungs.

## Introduction

DUSP3, or *Vaccinia* H1-Related (VHR), is a member of the atypical dual-specificity protein phosphatase family (A-DUSPs). This protein contains a 185 amino acids (Mr 21 kDa) catalytic domain and lacks targeting or docking domains [1]. Its shallow and broad catalytic pocket allows DUSP3 to dephosphorylate both p-tyrosine (p-Tyr) and p-threonine (p-Thr) residues [2]. The MAPKs ERK1/2, JNK and to a lesser extend p38 have been reported as substrates of DUSP3 [3–6]. The phosphatase also dephosphorylates other proteins such as the transcription factor STAT5 [7] and tyrosine kinase receptors EGFR and ErbB2 [8]. DUSP3 expression is regulated during cell cycle progression. Indeed its downregulation using RNA interference contributed to arrest HeLa cells in G1/S and G2/M phases and triggered their senescence. This was correlated with the hyper-phosphorylation of ERK1/2 and JNK [9]. Consequently, it is not surprising that overexpression of DUSP3 was found in human cervix carcinomas [10] and prostate cancer [11]. However, DUSP3 is also downregulated in other cancers such as breast cancer [12] and non-small cell lung carcinoma (NSCLC) [8,13], indicating contradictory and complex roles of DUSP3 in cancer development.

Recently, we generated full knock-out DUSP3-deficient (DUSP3^-/-^) mice by homologous recombination. These mice are viable, healthy and fertile, with no spontaneous phenotype. However, in these mice, DUSP3 deficiency prevented neo-angiogenesis and b-FGF-induced microvessel outgrowth [14].

In solid tumours, host cells such as endothelial cells, fibroblasts and immune cells represent a major part of cell populations within the tumour [15]. Macrophages are the most frequent immune cells in the tumour and their presence is mostly correlated with poor prognosis for the patient [16,17]. These macrophages are called tumour-associated macrophages (TAM) and regulate many, if not all stages of tumour progression from initiation of tumour development to metastatic dissemination including invasion, migration and extravasation processes [18]. Indeed TAM control the induction of angiogenesis, extracellular matrix remodelling, the stimulation of cancer cell proliferation and metastasis and the inhibition of anti-tumour immune responses [19]. TAM subpopulations are very heterogeneous and have different phenotypes. Hence, they can execute multiple and different functions related to tumour growth and reside in distinct areas within the tumour [20].

TAMs are shaped by factors secreted by tumour cells or by the tumour microenvironment to become immunosuppressive macrophages and exert pro-tumour responses. Indeed in addition to their trophic functions, TAMs are usually unable to lyse tumour cells, present tumour-associated antigens to T cells and express immune-stimulatory cytokines to stimulate the proliferation of anti-tumour functions of T cells and natural killer (NK) cells *in vitro*. Therefore, it has been proposed that TAMs are polarized into a M2-like phenotype (or alternatively activated-like macrophages) [21]. The molecular mechanisms responsible for this polarization are, however, poorly understood.

In this study, using a Lewis Lung carcinoma (LLC)-experimental metastasis model and DUSP3-deficient mice, we reported that the phosphatase DUSP3 is a key player in metastatic growth, modulating the recruitment of macrophages towards LLC-bearing lungs.

## Results

### DUSP3 deletion accelerates experimental LLC and E0771, but not B16, metastatic growth

**Fig 1:**
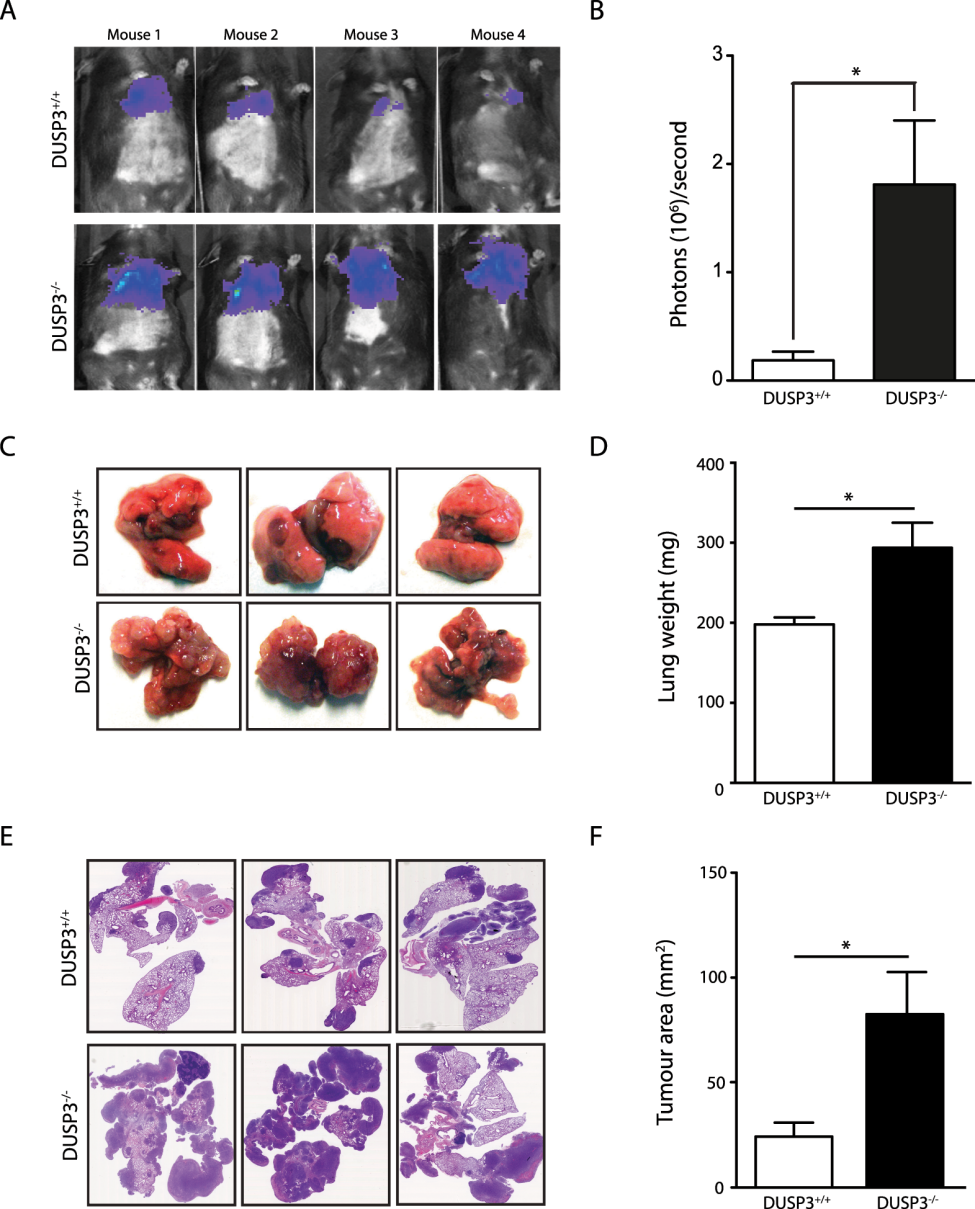
DUSP3 deletion accelerates experimental LLC metastasis growth. LLC tumour growths were monitored by xenogen bioluminescence imaging. Tumours were established by iv injection of 10^6^ LLC-Luc+ cells to DUSP3^+/+^ and DUSP3^-/-^ mice. **(A)** Representative xenogen imaging results. **(B)** Quantification of xenogen bioluminescence imaging data shown in A at day 14 after LLC injection. **(C)** Representative lung macroscopic view. **(D)** Comparison of lung weights from DUSP3^+/+^ and DUSP3^-/-^ mice. **(E)** Hematoxylin eosin staining of lung sections from DUSP3^+/+^ and DUSP3^-/-^ mice. **(F)** Comparison of tumour areas from DUSP3^+/+^ and DUSP3^-/-^ mice. Student t-test was used for (**B**) and (**D**) and Mann-Whitney test was used for (**F**). *p < 0,05, **p < 0.01. 4 mice were used in each group and for each experiment. Data shown are representative of 5 different experiments.

We have previously shown that DUSP3 plays an important role in tumour neo-vascularisation [14]. We showed that matrigel plugs and LLC subcutaneous tumours were less vascularized in DUSP3^-/-^ mice compared to DUSP3^+/+^ littermates. To investigate more in depth the roles of DUSP3 in experimental metastasis formation, we intravenously injected 1x10^6^ LLC-luciferase (LLC) cells into age and sex-matched DUSP3^+/+^ and DUSP3^-/-^ mice. LLC metastatic growth was followed *in vivo* by LLC cell luminescence signal quantification using the *in vivo* imaging system IVIS 200. Remarkably, the incidence of LLC lung metastasis was significantly higher in DUSP3^-/-^ compared to DUSP3^+/+^ mice (Fig 1A and 1B). At the time of sacrifice (day 14 after LLC injection), the DUSP3^-/-^ metastatic lung weight was significantly increased compared to DUSP3^+/+^ mice. Photographs of the lungs showed a major metastatic development in DUSP3^-/-^ lungs while only few nodules were visible in DUSP3^+/+^ mice (Fig 1C and 1D). Haematoxylin-eosin staining of lung sections and tumour area quantification confirmed that DUSP3^-/-^ lung tumours were significantly larger than in DUSP3^+/+^ lungs (Fig 1E and 1F).

**Fig 2:**
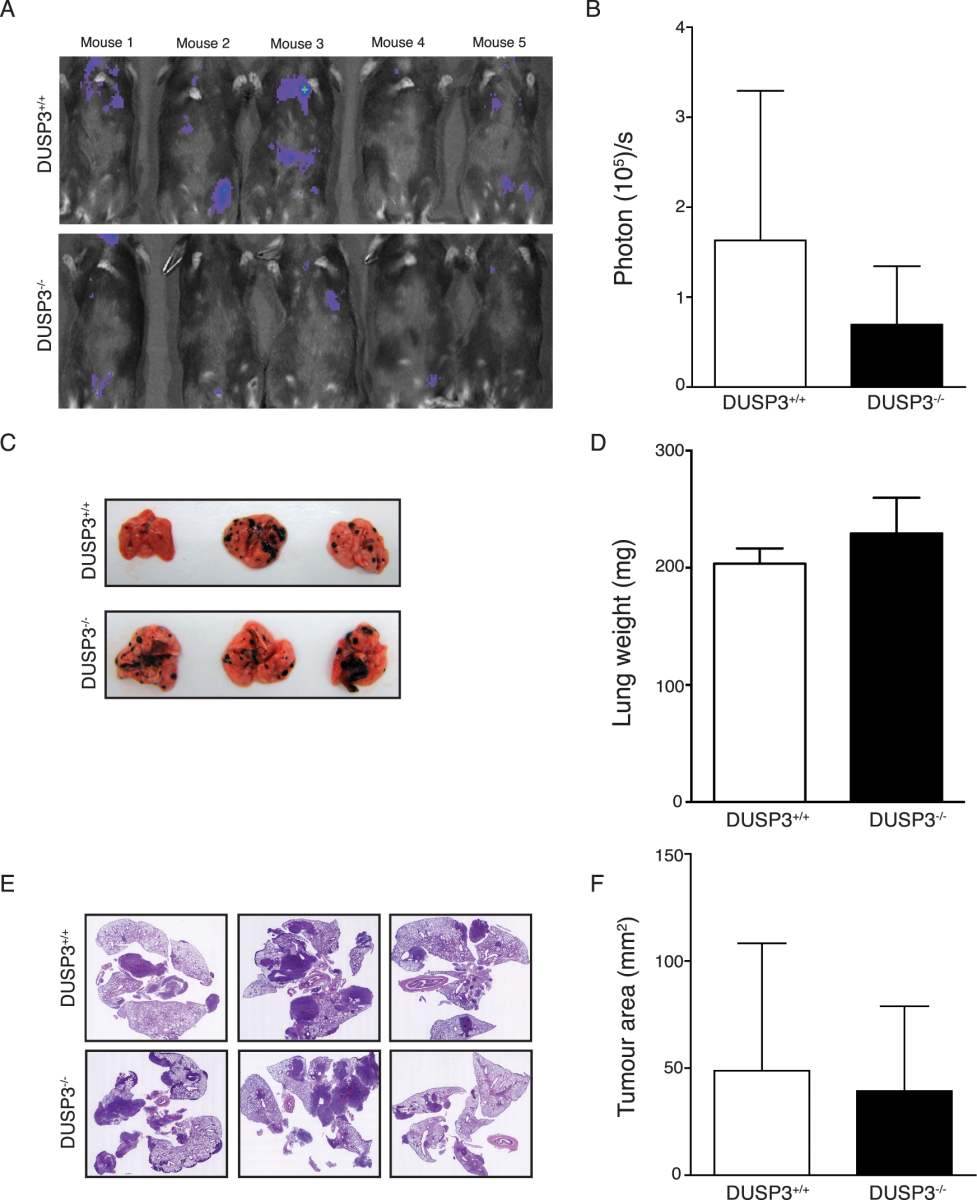
DUSP3 deletion does not impact experimental B16 metastasis growth. B16 tumour growths were monitored by xenogen bioluminescence imaging. Tumours were established by i.v. injection of 10^6^ B16-Luc+ cells to DUSP3^+/+^ and DUSP3^-/-^ mice. **(A)** Representative xenogen imaging results and **(B)** quantitative xenogen bioluminescence imaging data (day 14). **(C)** Representative lung macroscopic view and **(D)** comparison of lung weights from DUSP3^+/+^ and DUSP3^-/-^ mice. **(E)** Hematoxylin eosin staining of lung sections from each experimental group. **(F)** Comparison of tumour areas from each group. Student t-test was used for (**B**) and (**D**) and Mann-Whitney test was used for (**F**). *p < 0,05, **p < 0.01. 5 mice in each group were used for each experiment. Data shown are representative of 4 different experiments.

**Fig 3:**
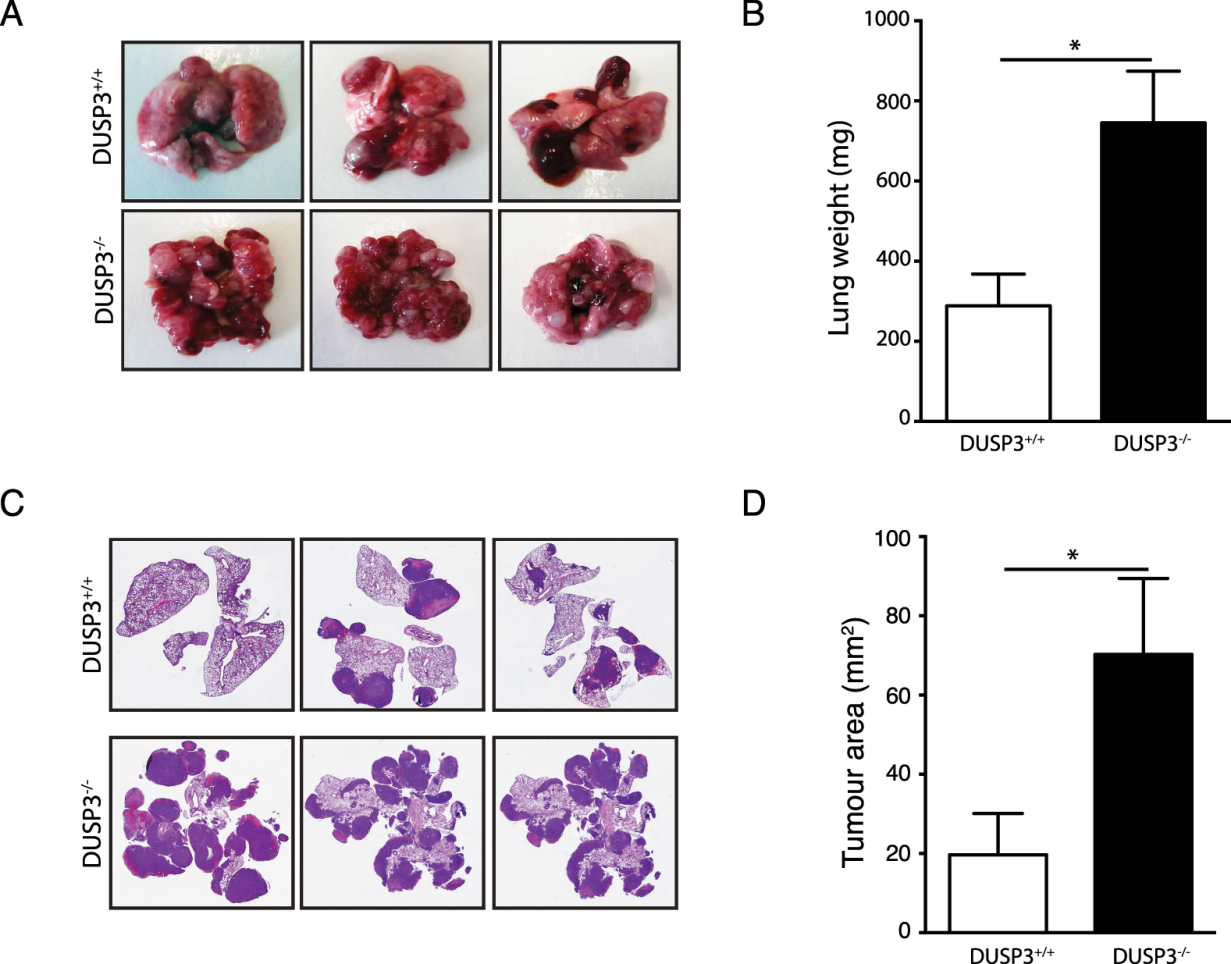
DUSP3 deletion accelerate experimental E0771 metastasis growth. E0771 tumours were established by i.v. injection of 1x10^6^ E0771 cells to DUSP3^+/+^ and DUSP3^-/-^ mice. **(A)** Representative lung macroscopic view at day 14 after injection. **(B)** comparison of lung weights from DUSP3^+/+^ and DUSP3^-/-^ mice. **(C)** Hematoxylin eosin staining of lung sections from each experimental group. **(F)** Comparison of tumour areas from each group. Student t-test was used for (**B**) and (**D**) and Mann-Whitney test was used for (**F**). *p < 0,05, **p < 0.01. 4 mice were used in each group and for each experiment. Data shown are representative of 2 different experiments.

To verify whether the marked increase of LLC growth in DUSP3^-/-^ mice was tumour model-dependent, we challenged DUSP3^+/+^ and DUSP3^-/-^ with two additional metastatic cells such as melanoma B16-F10-luciferase (B16) cells and E0771 cells. For B16, tumour growth was monitored using IVIS 200. Interestingly, there was no significant difference in the number and frequency of B16 metastatic foci between DUSP3^+/+^ and DUSP3^-/-^ mice. This was supported by the weight of B16-bearing DUSP3^+/+^ and DUSP3^-/-^ lungs and haematoxylin-eosin staining (Fig 2). Since E0771 cells do not express luciferase, tumour growth was evaluated at the time of sacrifice (14 days after cells injection) of the animals. Similarly to LLC cells, photographs of the lungs, weight of lungs, haematoxylin-eosin staining showed a significant metastatic development in DUSP3^-/-^ lungs while only few nodules were visible in DUSP3^+/+^ mice (Fig 3).

These observed differences suggest that the accelerated metastasis growth in DUSP3^-/-^ is tumour-model dependent. Since LLC and B16 cells are Luc+ and thus, facilitate *in vivo* monitoring of tumour growth, we decided to continue our investigations using these two cell lines only.

### LLC metastasis growth in DUSP3^-/-^ mice is driven by bone marrow derived cells

**Fig 4:**
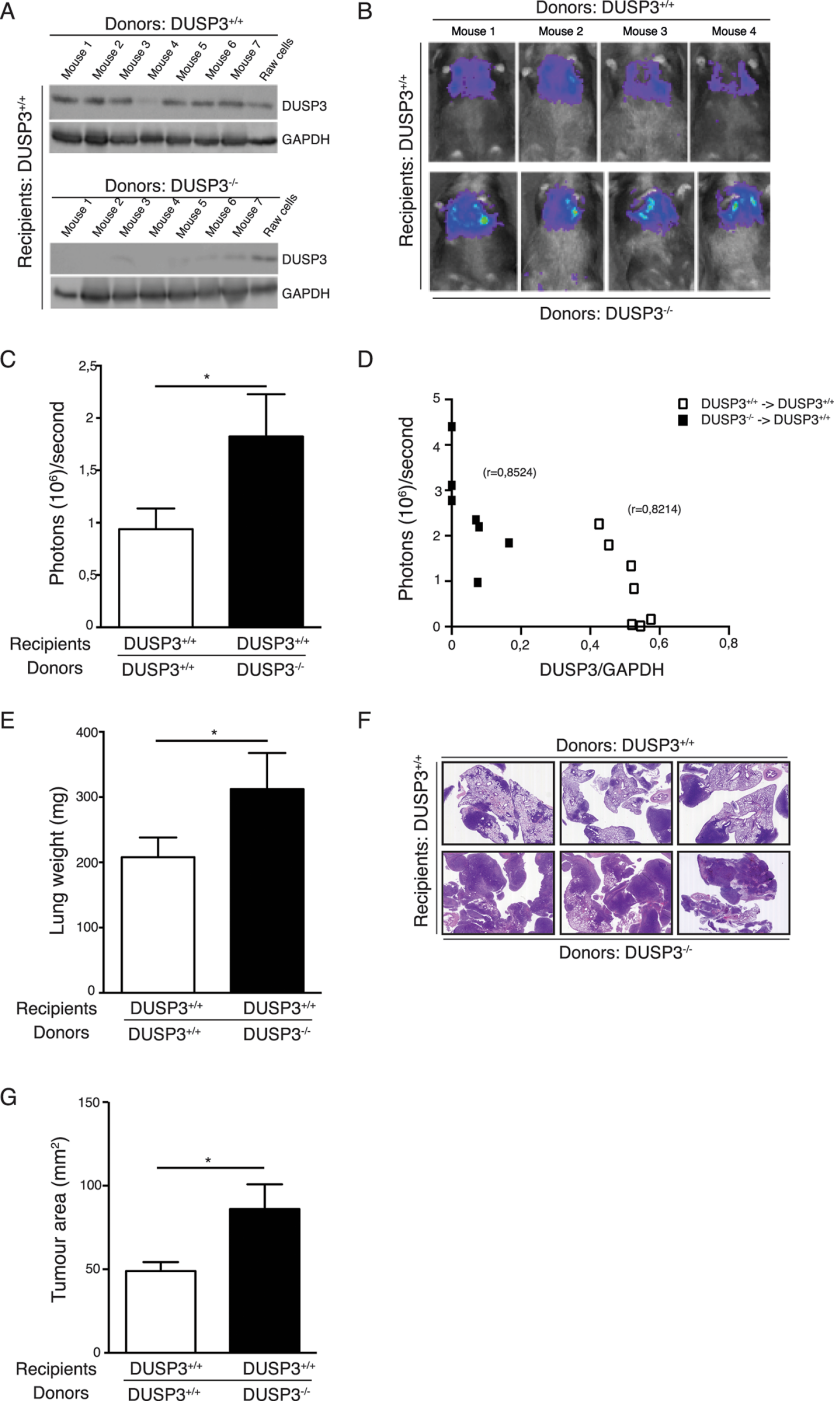
LLC metastasis growth in DUSP3^-/-^ mice is driven by hematopoietic cells. Tumours were established by injection (i.v.) of 10^6^ LLC-Luc+ cells to DUSP3^+/+^ or DUSP3^-/-^ BM-transplanted irradiated DUSP3^+/+^ mice. **(A)** Western blot analysis for DUSP3 expression from spleen lysates of transplanted mice (M = mouse). (**B**) Representative xenogen bioluminescence imaging results. **(C)** Quantitative imaging data (day 14). **(D)** Correlation between size of tumours (quantified as photons/second) and DUSP3 expression. **(E)** Comparison of lungs weights of DUSP3^+/+^ or DUSP3^-/-^ BM-transplanted irradiated DUSP3^+/+^ mice. **(F)** Hematoxylin-eosin staining of lung section from DUSP3^+/+^ or DUSP3^-/-^ BM-transplanted irradiated DUSP3^+/+^ mice. **(G)** Comparison of tumour areas from DUSP3^+/+^ or DUSP3^-/-^ BM-transplanted irradiated DUSP3^+/+^ mice. Student t-test was used for (**C**) and (**E**) and Mann-Whitney test was used for (**G**). *p < 0,05, **p < 0.01. 4 mice were used in each group and for each experiment. Data shown are representative of 2 different experiments.

Lung carcinomas are highly infiltrated with heterogeneous myeloid cell populations; among which some display a more tumour-promoting than others, although their precursors seem to be the same [22]. In a previous study, we showed that DUSP3 deletion in mice was associated with a polarization of macrophages towards the M2-like phenotype [23]. Since this cell population has been shown to play an important role in tumour promotion, we hypothesized that macrophages might be key actors in the enhanced LLC tumour cell dissemination observed in the LLC experimental model. Since DUSP3 knockout mice were generated using the standard homologous recombination method [14], we first generated chimeric mice by bone marrow (BM) transplantation of bone marrow cells from DUSP3^-/—^C57BL/6-CD45.2 mice to lethally irradiated DUSP3^+/+^-C57BL/6-CD45.1 mice (DUSP3^-/-^→DUSP3^+/+^ mice). Successful hemato-lymphoid reconstitution was verified by flow cytometry for the recipient mice 3–4 weeks after transplantation (data not shown) and by anti-DUSP3 immunoblot of spleen lysates at the day of sacrifice (Fig 4A). As a control, DUSP3^+/+^-C57BL/6-CD45.1 mice were transplanted with DUSP3^+/+^-C57BL/6-CD45.2 BM cells (DUSP3^+/+^→DUSP3^+/+^ mice). The obtained chimeric mice were then i.v. challenged with 1x10^6^ LLC cells. Lung metastasis development was monitored for 2 weeks after tumour cell injection, by measuring luciferase activity of tumour cells. Interestingly, DUSP3^+/+^ mice adoptively transferred with DUSP3^-/-^ bone marrow cells (DUSP3^-/-^→DUSP3^+/+^ mice) displayed an increased luciferase activity in lungs compared to DUSP3^+/+^→DUSP3^+/+^ transferred mice (Fig 4B and 4C). A significant correlation was measured between DUSP3 protein expression and the LLC bioluminescence (Fig 4D). Moreover lung weight and lung tumour area were significantly higher in DUSP3^-/-^→DUSP3^+/+^ mice compared to DUSP3^+/+^→DUSP3^+/+^ mice (Fig 4E–4G) suggesting that DUSP3-deficient hematopoietic cells contribute to enhance LLC tumour aggressiveness (Fig 4).

**Fig 5:**
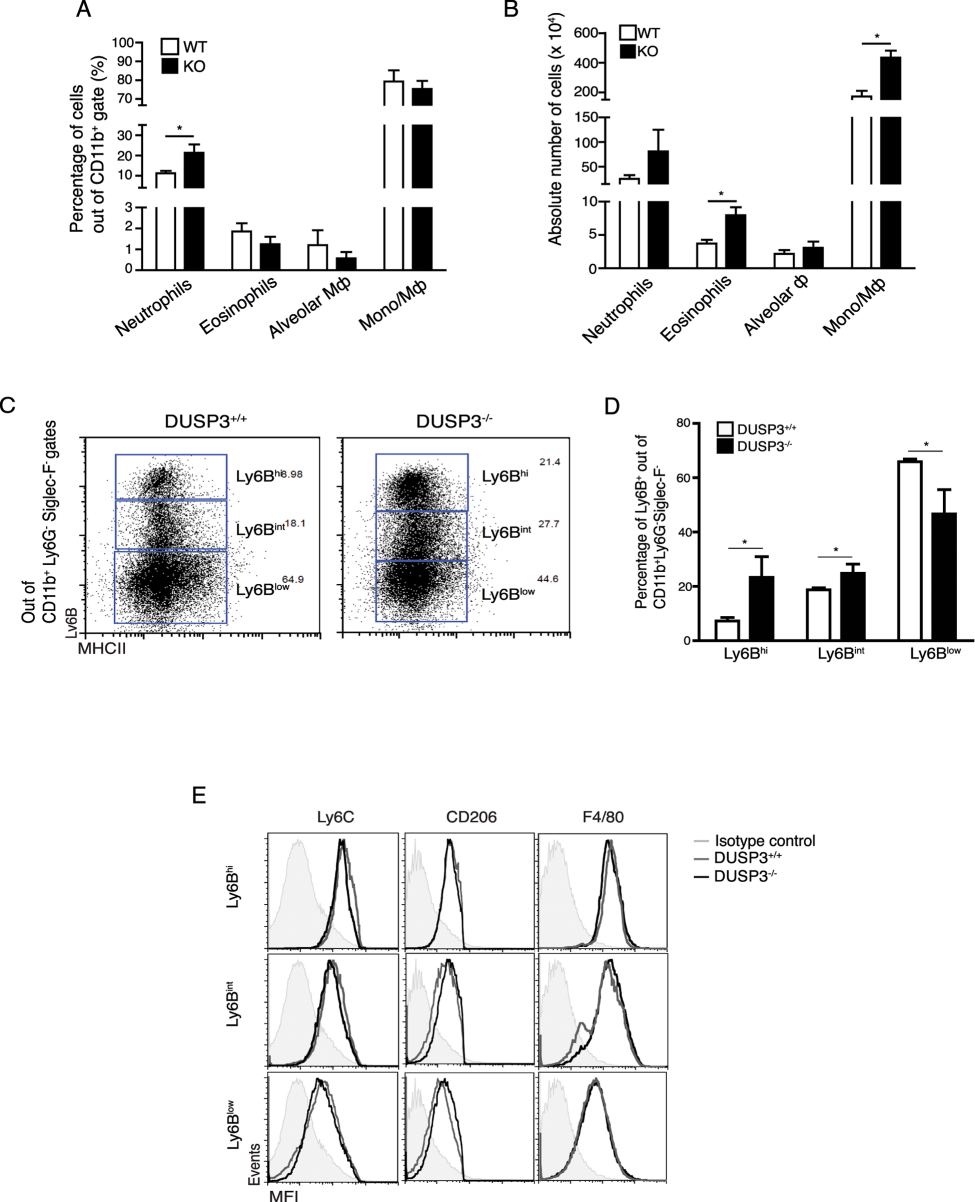
LLC metastasis growth in DUSP3^-/-^ mice is associated with the presence of higher monocytes and macrophages in lungs and with increased proliferation. Immune cell population phenotyping in LLC tumour-bearing lungs by flow cytometry. **(A)** Percentages of neutrophils, eosinophils, alveolar macrophages and monocytes/macrophages in DUSP3^+/+^ and DUSP3^-/-^ lungs. **(B)** Absolute number of neutrophils, eosinophils, alveolar macrophages and monocytes/macrophages in DUSP3^+/+^ and DUSP3^-/-^ lungs. **(C-E)** Monocytes/macrophages phenotype characterization in LLC tumours: Comparison **(C)** and percentage **(D)** of Ly6B^hi^, Ly6B^int^ and Ly6B^low^ macrophages in DUSP3^+/+^ and DUSP3^-/-^ mice. **(E)** Expression of specific macrophages markers in distinct Ly6B^+^ populations. Alveolar Ф = alveolar macrophages; Mo/MФ = monocytes/macrophages. Student-t-test was used for statistical analysis. *p < 0,05. 3 mice were used in each group and for each experiment. Data shown are representative of 4 different experiments.

To further assess which hematopoietic cell populations could be involved in the increased tumour aggressiveness, we analysed, in first instance, by flow cytometry, the myeloid cell subsets present in established DUSP3^+/+^ and DUSP3^-/-^ lung homogenates bearing LLC tumours. The gating strategy is described in S1 Fig. Briefly, after gating on CD45^+^ and CD11b^+^ cells to select myeloid cells, neutrophils, eosinophils and alveolar macrophages were discriminated based on Ly6G and Siglec-F surface markers. Macrophages/monocytes were considered Ly6G^-^siglecF^-^ and with various expression of Ly6C and MHC-II as previously reported [24]. The percentage of CD45.2 and CD11b positive cells was higher in DUSP3^-/-^lung homogenates compared to DUSP3^+/+^ (data not shown). The percentage of neutrophils was significantly higher in DUSP3^-/-^ lungs compared to DUSP3^+/+^. On the contrary, the percentage of eosinophils, alveolar macrophages and monocytes remained unchanged in DUSP3^+/+^ and DUSP3^-/-^ lungs. However, the absolute number of eosinophils and monocytes/macrophages were significantly higher in DUSP3^-/-^ lungs compared to DUSP3^+/+^. The number of neutrophils and alveolar macrophages was slightly but not significantly elevated in DUSP3^-/-^ compared to DUSP3^+/+^ lungs (Fig 5A and 5B). We next characterized the different monocyte/macrophage subpopulations within LLC-bearing lungs based on Ly6C and MHC-II expression together with CD206, F4/80 and Ly6B markers. In DUSP3^+/+^ metastatic lungs, only 10% of macrophages were Ly6B^hi^, while about 20% and 65% of cells were Ly6B^int^ and Ly6B^low^, respectively (Fig 5C and 5D). On the opposite, the percentage of Ly6B^hi^ and Ly6B^int^ macrophages were significantly increased in DUSP3^-/-^ mice with more than 20% and 30% among the MHC-II positive cells, respectively. The DUSP3^+/+^ Ly6B^low^ cells were significantly decreased compared to DUSP3^+/+^ cells (Fig 5D). The expression of F4/80, Ly6C and CD206 on these populations confirmed the identity of macrophages (Fig 5E). As a control, Ly6B expression was measured on B16-tumor bearing lungs but no difference was found between DUSP3^+/+^ and DUSP3^-/-^ macrophages (supplemental S2 Fig).

**Fig 6:**
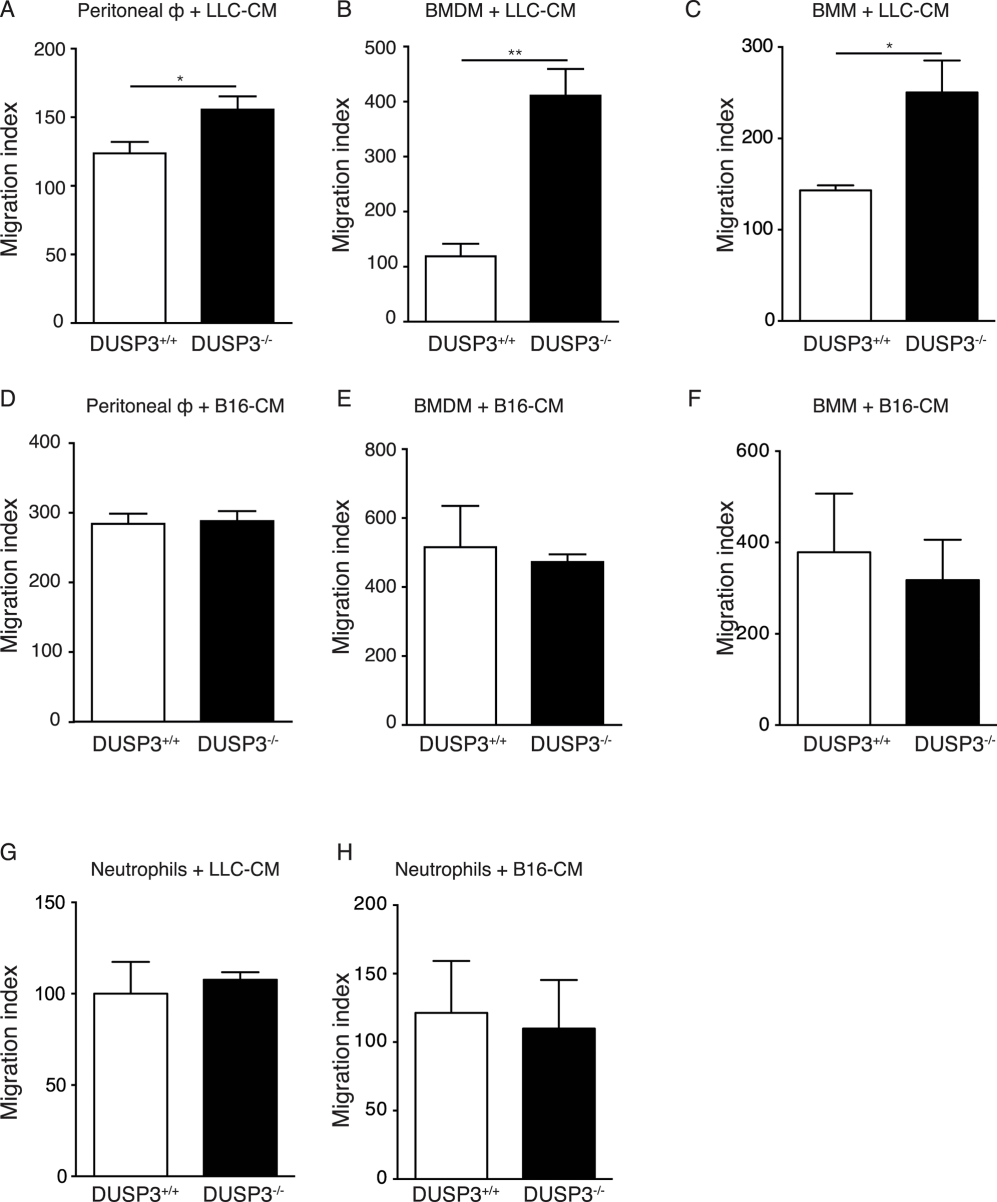
DUSP3^-/-^ monocytes and macrophages migration is enhanced in response to LLC-conditioned medium. *In vitro* migration assay of DUSP3^+/+^ and DUSP3^-/-^ peritoneal macrophages **(A),** BMDMs **(B)** and BMMs **(C)** in presence of LLC-CM. Migration of DUSP3^+/+^ or DUSP3^-/-^ peritoneal macrophages **(D)**, BMDMs **(E)** and BMMs **(F)** in presence of B16-CM. Migration of DUSP3^+/+^ or DUSP3^-/-^ bone marrow sorted neutrophils in presence of LLC-CM **(G)** and B16-CM **(H**). BMDM = Bone Marrow-Differentiated Macrophages; CM = conditioned-medium. Student-t-test was used for statistical analysis. *, p < 0,05 and **, p < 0.01. For each experiment, cells were pooled from 3 mice. Data shown are representative of 3 different experiments.

A potential explanation for the higher presence of recently migrated Ly6B^hi^ cells in DUSP3^-/-^ mice could be that soluble factors secreted by LLC enhance the migration of these cells. We therefore performed an *in vitro* migration assay of DUSP3^-/-^ and DUSP3^+/+^ bone marrow sorted monocytes (BMM), peritoneal macrophages (PM) and bone marrow-derived macrophages (BMDM) in response to LLC-conditioned medium (LLC-CM). For PMs and BMDMs, after 18h migration, both DUSP3 deficient cells migrated significantly better in response to LLC-CM compared to DUSP3^+/+^ derived macrophages (Fig 6A and 6B). For BMMs, differences were similar and already visible and significant after only 1h 30 min of migration (Fig 6C). As a control, we performed the same *in vitro* migration assay in response to B16-conditioned medium (B16-CM). On the contrary to LLC-CM, B16-CM did not influence differently the migration of DUSP3^+/+^ and DUSP3^-/-^ BMM, PM and BMDMs (Fig 6D, 6E and 6F). To investigate if this difference in migration was restricted to monocytes and macrophages or a general feature of DUSP3^-/-^ cells, we performed the same migration assay on bone marrow neutrophils from and observed no difference in migration of these cells between cells from DUSP3^+/+^ and DUSP3^-/-^ in all culture conditions (Fig 6G and 6H).

### Macrophage depletion reduces LLC tumour growth in DUSP3^-/-^ mice

**Fig 7:**
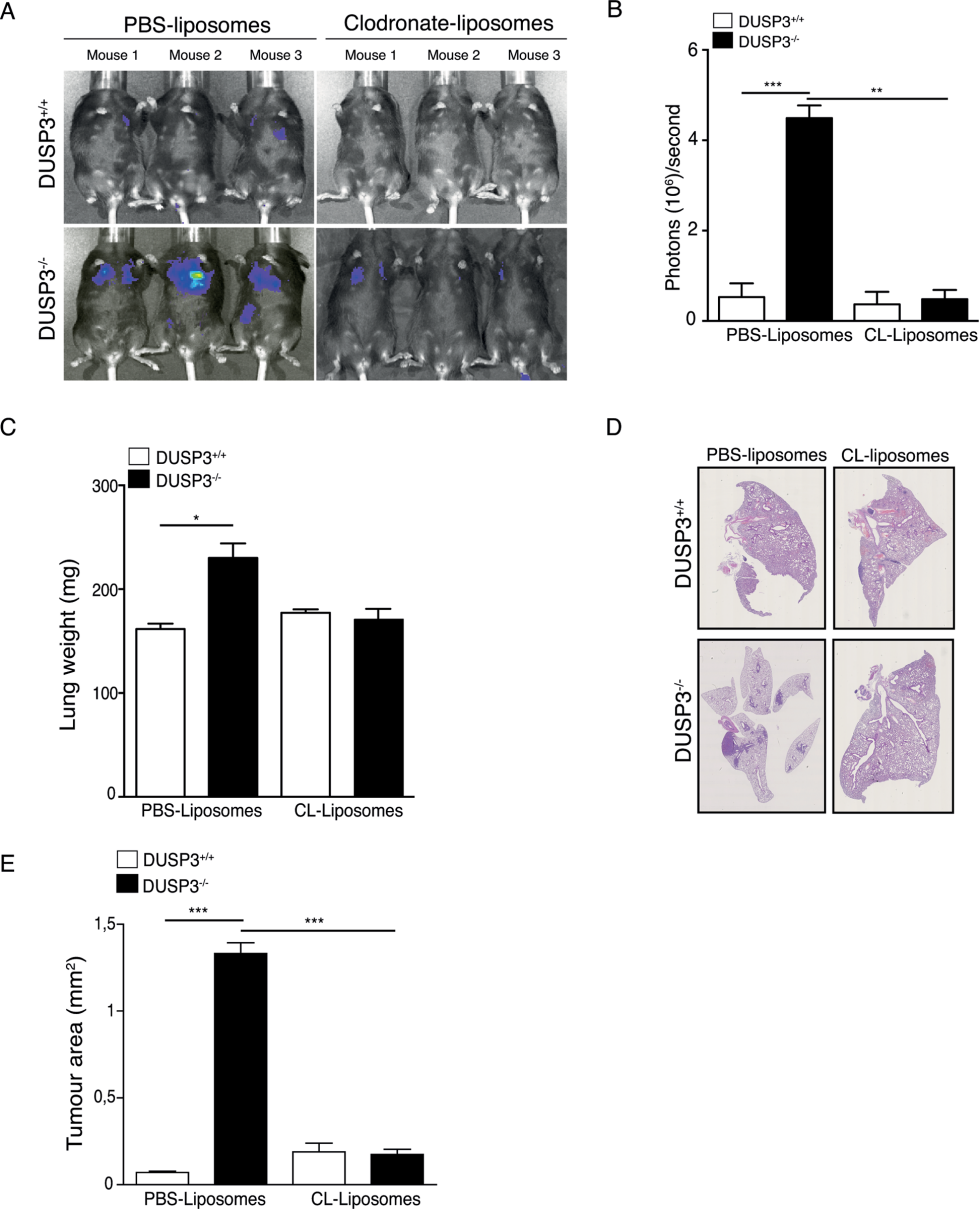
Macrophages depletion reduces LLC tumour growth in DUSP3^-/-^ mice. Tumours were established by i.v. injection of 10^6^ LLC-Luc+ cells to clodronate-liposomes-depleted mice. **(A)** Representative xenogen imaging results and **(B)** quantification of the xenogen bioluminescence imaging data shown in A at day 14 after LLC injection. **(C)** Comparison of lung weights. **(D)** Representative hematoxylin eosin staining of lung sections from DUSP3^+/+^ and DUSP3^-/-^ mice injected with PBS-lipsomes or clodronate-liposomes. **(E)** Comparison of tumour areas from each group. Student-t-test was used for statistical analysis. *p < 0.05, **p < 0.01 and ***p < 0.001. 3 mice were used in each group and for each experiment. Data shown are representative of 3 different experiments.

Together, the obtained data suggest that myeloid cells, and in particular macrophages, accelerate LLC lung tumour progression by a mechanism involving the phosphatase DUSP3. To confirm this finding, we chemically depleted macrophages in DUSP3^+/+^ and DUSP3^-/-^ mice, using clodronate-liposomes. Mice were first intravenously and intraperitoneally injected with clodronate-liposomes or with empty-liposomes as control. 48 hours after the first injection of liposomes, mice were intravenously injected with 1x10^6^ LLC cells, after which the intraperitoneal injection of clodronate-liposomes was repeated every other day and up to two weeks after LLC challenge. Finally, the lung metastasis formation was monitored, using in vivo imaging. The efficiency of macrophage depletion was verified at the day of sacrifice by comparing the presence of residual macrophages in both peritoneal exudates and lung cell suspensions of empty-liposome versus clodronate-liposome injected mice. In peritoneal exudates, CD11b^+^-F4/80^+^ macrophages completely disappeared after clodronate-liposome injection. In lung homogenates, one population of Ly6B^hi^ macrophages was significantly reduced after clodronate-liposome injection compared to control conditions (S3 Fig). Compared to vehicle liposome-treated mice, elimination of macrophages decreased significantly LLC metastatic dissemination in both DUSP3^+/+^ and DUSP3^-/-^ mice as demonstrated by the decreased bioluminescence (Fig 7A and 7B), weight (Fig 7C) and tumor area in lungs (Fig 7D and 7E). Interestingly, in the absence of macrophages, no significant difference could be observed in LLC metastasis growth between DUSP3^-/-^ and DUSP3^+/+^ mice (Fig 7). These data indicate that DUSP3-deficiency in monocytes and macrophages accelerates metastatic growth.

### Increased infiltration of macrophages in DUSP3^-/-^ mice lungs is associated with increased in situ proliferation of LLC cells

**Fig 8:**
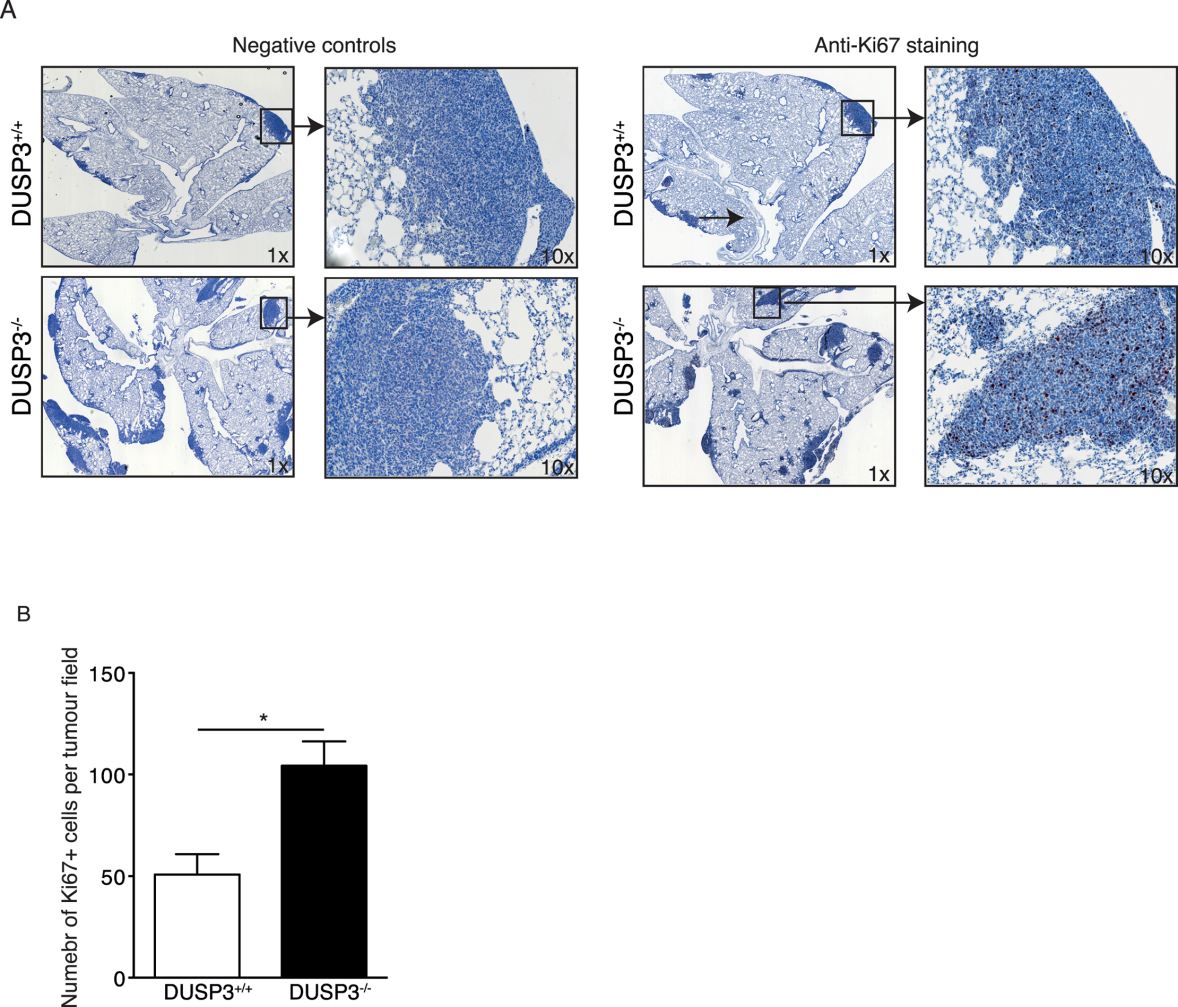
Increased LLC tumour mass in DUSP3^-/-^ mice lungs is associated with increased in situ proliferation of these cells. Sections of 5-μm thickness were cut from DUSP3^-/-^ and DUSP3^+/+^ LLC bearing lungs embedded in paraffin blocks. Immunohistochemistry for Ki67 was carried out. Revelation was performed using AEC+ Red. Negative control sections were traited similarly except that they were not stained with anti-KI67. **(A)** repesentative images from each group of mice are shown at 1x and 10x magnifications. Dark color indicate positive staining for Ki67. **(B)** Quantification of the positively stained cells from the entire tumor represented in the section using Image J software. Student-t-test was used for statistical analysis. *p < 0.05. Data shown are representative of 5 different sections scanned from 5 individual mice from each genotype.

Several studies have demonstrated that the density of TAMs positively correlates with tumor growth. Although the mechanisms are poorly understood, it has been demonstrated that TAMs secrete several factors that contribute to immunosupresion, stimulate tumor cells proliferation, promote angiogenesis and metastasis [18][19][25][26]. To investigate the impact of the observed increased infiltration of macrophages in LLC, we hypothesised that DUSP3^-/-^ macrophages may increase the *in situ* proliferation of LLC cells. We therefore quantified proliferating tumour cells using anti-Ki67 on lungs sections from DUSP3^-/-^ and DUSP3^+/+^ tumours bearing mice. We found that Ki67^+^ cells number was significantly higher inside tumour zones of lungs from DUSP3^-/-^ compared to DUSP3^+/+^ mice (Fig 8). These data suggest that the observed increased tumour mass in DUSP3^-/-^ mice lungs is at least partially due to *in situ* enhanced proliferation of LLC cells.

## Material and methods

### Antibodies and reagents

Purified anti-CD16/CD32 (FcγIII/II receptor) (2.4G2), PE-anti-Ly6G (1A8), APC-Cy7 anti-Ly6G (1A8), V450-anti-CD45.2 (A20), V500-anti-I-A/I-E (MHC-II) (M5/114.15.2), APC-anti-CD11c (HL3), APC-Cy7 anti-CD11c (HL3), PE anti-Siglec-F (E50-2440), PE-Cy7-streptavidin and Biotin anti-IgG2B (RG/11.1) were from BD Biosciences (BD Biosciences, San Jose, CA). APC-anti-F4/80 (BM8) and PerCp-Cy5-anti-CD11b (M1/70) were from eBiosciences (eBioscience, San Diego, CA). Alexa 647-anti-CD206 (C068C2) and PE-Cy7-anti F4/80 (BM8) were from Biolegend (Biolegend, San Diego, CA). Alexa647 anti-Ly6b.2 (7/4) and Alexa 488-anti-Ly6G (ER-MP20) were from AbD Serotec (AbD Serotec, Kidlington, UK). Anti-VHR (DUSP3) (sc8889) antibody was from Santa Cruz (Santa Cruz, Dallas, Texas). Anti-GAPDH antibody was from Sigma (Sigma-Aldrich, Diegem, Belgium). Anti-Ki67 antibody was from Abcam (Abcam, Cambridge, UK). HRP-conjugated anti-goat, anti-mouse and anti-rabbit were used as secondary antibodies and were from Amersham Biosciences (Amersham Biosciences, GlattBrugg, Switzerland). Collagenase I and DNaseI were from Roche (Roche, Basel, Switzerland)

### Mice and ethic statement

C57BL/6 (CD45.2)-DUSP3^-/-^ mice were generated at the University of Liege SPF animal facility as previously reported [14]. These mice were backcrossed with C57BL/6-CD45.2 mice from Charles River (Charles River) to generate heterozygotes that were mated to generate DUSP3^+/+^ and DUSP3^-/-^ littermate colonies used for experimentation. Age matched female DUSP3^+/+^ and DUSP3^-/-^ mice were used in all the experiments. Mice were kept in ventilated cages under 12-hour dark/12-hour light cycle in the University of Liege SPF animal facility and received food and water and libitum. Health status was evaluated every 3 months and mice were always found free of specific pathogens.

All mice experiments and procedures were carried out following the guidelines of and in agreement with the animal ethics committee of the University of Liège (agreement Number: 858). All efforts were made to minimize animal suffering. Animals were monitored continuously for signs of distress or pain by FELASA-qualified personal (categories B and C) in accordance with the European guidelines for the care and maintenance of laboratory animals. After tumour cells injection, monitoring for animal health was performed every day until the mice sacrifice at day 14 post-injection. Animals were weighted twice a week. *In vivo* xenogen imaging was used to evaluate tumour development and progression and was performed twice, at day 7 and 14 after cells injection. Since tumours were internal, we were not able to measure the progression of tumour size. Assessment of severity was evaluated by a monitoring scheme including careful observation of any abnormal animal posture, locomotion, activity, behavior and weight loss exceeding 20%. During this study none of the animals displayed symptoms of suffering or reached the termination criteria (weight loss > 20%, hunched posture, decreased activity/locomotion, agitation and abnormal aggressiveness).

### Tumour metastasis model

LLC cells (1x10^6^ cells) or B16 cells (1x10^6^ cells) were inoculated to 8–12 weeks old DUSP3^+/+^ and DUSP3^-/-^ mice on day 0 via tail vein injection. Tumour development was examined by xenogen imaging at 7 and 14 days after cells injection and mice were sacrificed on day 14. E0771 cells (1x10^6^) were inoculated to 10 weeks old DUSP3+/+ and DUSP3-/- mice via tail vein injection. Mice were sacrificed on day 28. LLC-, B16- and E0771-bearing lungs were photographed and weighted before fixation for 2h in 4% paraformaldehyde or preparation of single-cell suspensions.

### Xenogen imaging

The LLC or B16 tumour progression was monitored using Imaging System Xenogen IVIS 200 (Advanced Molecular Vision, Caliper Lifesciences, Waltham, MA, United States). 100 μl of luciferin potassium salt (30mg/ml) was intraperitoneally injected in tumour bearing DUSP3^+/+^ and DUSP3^-/-^ mice. After 12 minutes, the photons emitted by the luciferase activity were detected. The bioluminescence was quantified using the Living Image Software (Caliper Life Sciences, Waltham, MA, United States) and by delimitation of region of interest (ROI) around the lungs.

### Mice irradiation and bone marrow transplantation

10 to 12 weeks old donor mice were sacrificed by cervical dislocation. Tibias and femurs were collected, separated and cleaned in sterile PBS on ice. Bone marrow cells (BMs) were flushed using a 25-gauge needle and 1 mL syringe filled with cold PBS. BM cell suspensions were passed through 70 μm nylon cell strainer (BD biosciences, San Jose, CA, United States) and centrifuged 10 min at 1200 rpm at 4°C. Single cells were then counted and resuspended in PBS at a concentration of 10x10^6^ cells/150 μL on ice. Cells were immediately transplanted to 6 weeks old lethally irradiated (849,1 cGy) recipient mice, via intravenous (i.v) injection. The efficiency of the transplantation was assessed by Western blot using anti-DUSP3. Anti-GAPDH immunoblot was used for normalization.

### Macrophage depletion

150 μL of clodronate-liposome or empty-liposome (ClodronateLiposomes, Haarlem, The Netherlands) were injected intravenously and intraperitoneally in DUSP3^+/+^ and DUSP3^-/-^ mice, 2 days prior to LLC injection. The intraperitoneal injections were repeated every other day during 14 days. The efficiency of the depletion was assessed by flow cytometry on peritoneal washes and lung cell suspensions.

### Isolation of Bone Marrow Derived monocytes and in vitro differentiation of macrophages

Bone marrow (BM) cells were aseptically flushed out from femurs and tibias of DUSP3^+/+^ and DUSP3^-/-^ mice on ice, using a 25-gauge needle and FBS-free RPMI. The cell suspensions were filtered using a 100 μm cell strainer and centrifuged 10 min at 1200 rpm at 4°C. Monocytes were then negatively sorted using EasySep Mouse Monocyte isolation kit (STEMCELL technologies, Vancouver, Canada), according to manusfacturer’s instructions. Sorted cells were next directly used for migration assay. For in vitro macrophages differentiation, 25x10^6^ of BM cell suspension were plated in 12 mL of RPMI supplemented with 10% heat-inactivated FBS, 10% L929-conditionned medium, streptomycin (100μg/mL) and penicillin (100U/mL) for 7 days. The cells were washed and the medium changed every other day. The macrophage differentiation was assessed by a F4/80-CD11b flow cytometry staining.

### Isolation of peritoneal macrophages

Resident peritoneal macrophages (PMs) were selected by adherence to tissue culture plastic dishes in culture conditions at a cell density of 1 3 106 cells /mL in complete RPMI 1640 medium. After 2h, cells were gently rinsed twice with FBS/RPMI 1640 and used for experiments.

### Cell migration

The bone marrow derived macrophages (BMDM) and monocytes migration was assessed using 5 μm polycarbonate transwells (Corning, Lowell, MA). The membrane was equilibrated for 1h at 37°C with 600 μL and 100 μL of DMEM in the lower chamber and in the upper chamber, respectively. 3x10^5^ cells were plated in 100 μL of medium in the upper chamber while 600 μL of DMEM, LLC- or B16- conditioned-medium was loaded in the lower chamber. Macrophages and monocytes were let to migrate for 18h and 1h30min respectively at 37°C. The cells that migrated to the lower chamber were recovered and counted using the Millipore ScepterTM cell counter after gating on live cells using the Scepter Software 1.2 (Millipore, Overijse, Belgium). Migration index was calculated as number of cells that transmigrated in the presence of chemokine per number of cells that transmigrated in the absence of the chemokine multiplied by 100.

### Lung histology and immunohistochemistry staining

Lungs were fixed in 4% paraformaldehyde, paraffin embedded, cut in 5-μm sections, and stained with haematoxylin and eosin. 5 randomly selected sections per mouse lungs were analysed. Tumour areas were quantified using NanoZoomer Digital Pathology Image software (Hamamatsu, Japan). For immunohistochemical detection of Ki67 in lung tumours, sections of 5-μm thickness were cut from paraffin blocks and immunohistochemistry for Ki67 (dilution 1:500, incubation time: 30min at room temperature) was carried out using Dako Envision+ System—HRP Labeled Ploymed anti-Rabbit (Dako K4003). Revelation was performed using AEC+ Red (Dako K3461). Stained slides were next scanned using NanoZoomer Digital Pathology whole slide scanner (Hamamatsu, Japan). Only distinct nuclear staining was used for quantification Assessment was carried out on the entire tumor represented in the section using Image J software.

### Cell culture

Lung Lewis carcinoma cells, stably transfected with luciferase gene (LL/2-luc-M38, LLC) were cultured in Dulbecco’s modified Eagle’s medium (DMEM) (Lonza, Basel, Switzerland) supplemented with 10% heat-inactivated foetal bovine serum (FBS), penicillin (100U/mL), streptomycin (100ug/mL) and neomycin (G418, 0,02mg/mL) (life technologies, Carlsbad, CA, United States). The B16 melanoma cell line (B16-F10-luc-G5, B16), stably transfected with luciferase gene, was maintained in culture in RPMI (Lonza, Basel, Switzerland) supplemented with 10% heat-inactivated FBS and penicillin (100U/mL), streptomycin (100ug/mL) and zeocyn (0,2mg/mL) (life technologies, Carlsbad, CA, United states). LLC and B16 cells were purchased at Xenogen/Caliper.

### Western blot

Cells were lysed using RIPA buffer (50 mMTris-HCl (pH = 8.0), 150 mM NaCl, 1% NP-40, 0.5% sodium deoxycholate, 0.1% SDS, 1 mM orthovanadate, complete protease inhibitor cocktail tablets EDTA free and 1 mM phenylmethylsulfonyl fluoride) on ice during 20 minutes. Lysates were next clarified by centrifugation at 21.000 g during 20 min at 4°C. The resulting supernatants were collected and protein concentrations were determined using the colorimetric Bradford reagent (Bio-Rad, Nazareth, Belgium). Samples were next denaturated at 95°C in Laemmli buffer. Samples were then run on SDSPAGE gel and transferred to Hybond-nitrocellulose membranes. To block the non-specific binding sites, membranes were incubated for one hour in Tris-buffered saline-Tween 20 containing 5% of non-fat milk. The membranes were next immunoblotted with anti-DUSP3 antibody. Membranes were next stripped, blocked and immunoblotted with anti-GAPDH, antibody for normalization. Immunoreactivity was then revealed using HRP conjugated secondary antibodies. The blots were developed by enhanced chemiluminescence (Amersham, Gent, Belgium) according to the manufacturer’s instructions.

### Preparation of single-cell suspensions from lungs

Lungs were perfused with 5mL PBS through the right ventricle, then dissected and shopped into small pieces before digestion for 1h at 37°C in 4mL of HBSS 1x containing 1 mg/mL collagenase A, 0.05 mg/mL DNaseI and 5% FBS. Mechanical stress was applied to the cells by flushing them through 18 gauge-needle. The cells were then passed through a 70 μm nylon cell strainer, centrifuged for 7 min at 1400 rpm at 4°C and the red blood cells were lysed. Isolated cells were directly stained for flow cytometry analysis.

### Flow cytometry and phenotyping

For surface cell staining, 5x10^5^ to1x10^6^ cells were incubated for 15 minutes with anti-CD16/CD32 (FcγIII/II receptor) using 0.5μg/100μL concentration to block non-specific interactions, prior to labelling for 30 minutes with specific antibodies. All stainings were performed on ice in PBS followed by one washing in PBS. Cells were next analysed on FACS Canto II (Becton Dickson, San Jose, CA, United States). Analysis was done using Flowjo (Flowjo, Ashland, Or, United States)

### Statistical analysis

Statistical analyses were performed using Prism software (GraphPad, San Diego, CA, United States). The “D’Agostino and Pearson omnibus normality test” was used to assess the normality of the distribution. Depending on the distribution, the student T-test or Mann Whitney test were applied to determine the difference between two experimental conditions. All the results are presented as mean ± SEM. The results were statistically significant when p-value <0,05. *p<0,05; **p<0,01; ***p<0,001.

## Discussion

Metastasis is the primary cause of death in cancer patients. The mechanisms underlying metastasis development are however not yet completely understood. Several DUSPs have been associated with metastasis formation and their expression/activity correlates with poor clinical outcome [27,28]. Most scientific studies investigating the roles of DUSPs in metastasis formation focused on tumour themselves, while only few studies analysed the roles of DUSPs in the tumour microenvironment [29]. Using a recently developed DUSP3 full-knockout mouse strain and a model of experimental LLC-metastasis, shortcutting primary tumour growth and intravasation processes, we report that DUSP3 deficiency favours LLC-induced macrophage recruitment at the tumour site, thus enhancing pulmonary metastasis formation. This study highlights a new role for DUSP3 in the susceptibility to develop lung metastasis.

In the present work, we demonstrate that LLC-lung metastasis formation is enhanced in DUSP3^-/-^ mice compared to DUSP3^+/+^ littermates. This phenomenon was however specific to LLC and E0771 cells since no difference of B16 metastatic dissemination was observed between DUSP3^+/+^ and DUSP3^-/-^ mice. Studies have reported that reduced vascular permeability led to a decrease in metastasis after LLC and B16 i.v. injection [30,31]. Although vascular permeability is significantly enhanced in DUSP3^-/-^ mice when compared with DUSP3^+/+^ mice (unpublished observations), the fact that B16-induced metastasis was not influenced by DUSP3 deletion rules out the possible involvement of vascular permeability in the observed phenotype. The second plausible premise could be that LLC and E0771-metastatic cells respond differently to the DUSP3^-/-^ tumour microenvironment. A differential regulation of experimental LLC and B16 metastasis formation has been previously shown in Nrf2-deficient mice. Indeed these mice developed lung metastasis faster compared to control mice upon LLC but not B16 cells i.v. injection [32]. In the case of LLC metastasis, tumour formation was associated with a higher recruitment of immune cells (MDSC). The authors of the study concluded that Nrf2 facilitates appropriate immune responses against LLC cells and therefore plays an anti-metastatic role and that the phenotype was restricted to the lung microenvironment [32]. The differential involvement and regulation of immune cells was further confirmed by the fact that the recruitment of myeloid-derived suppressor cells and dendritic cells are different in LLC and B16 experimental metastasis [33]. In our model, the role of immune cells was clearly demonstrated by the use of bone marrow chimeric mice. The transplantation of DUSP3^-/-^ bone marrow cells into irradiated DUSP3^+/+^ mice accelerated the development of metastatic in recipient mice since DUSP3^-/-^ -> DUSP3^+/+^ mice developed significantly larger lung metastasis compared to control mice, demonstrating that the hematopoietic compartment is responsible for the increased LLC metastasis in DUSP3^-/-^ mice.

We recently reported that DUSP3 plays an important role in innate immunity and most precisely in macrophages. Tumour-associated macrophages (TAM) display in general M2-like properties, although a high diversity exists [25]. Based on this knowledge, macrophages could play a role in the present LLC metastasis model by creating a tumour-sustaining environment. Specific elimination of macrophages using clodronate-liposomes decreased LLC-metastasis in both DUSP3^+/+^ and DUSP3^-/-^ mice. More importantly, macrophage depletion abolished the difference of lung tumour growth between DUSP3^+/+^ and DUSP3^-/-^ mice. Moreover, in this experiment, we did not observe any decrease of neutrophil percentage, as verified by flow cytometry staining. This experiment rules out the involvement of neutrophils in the observed phenotype, despite the fact that a higher percentage of these cells was found in LLC-bearing lungs from DUSP3^-/-^ mice. It would be interesting to investigate the significance of such increase and the exact role of neutrophils in LLC-metastasis formation in a DUSP3-dependent manner.

In a subcutaneous model of LLC tumour growth, Movahedi et al. showed that distinct subsets of monocytes/macrophages existed inside the tumour. They characterized the different monocyte/macrophage populations based on the expression of CD11b, Ly6C and MHC-II surface markers [24]. We performed a similar analysis on tumour-bearing lung cell suspensions of DUSP3^+/+^ and DUSP3^-/-^ mice. Phenotypically, DUSP3^+/+^ and DUSP3^-/-^ monocytes/macrophages did not differ from each other. However, using the additional marker Ly6B, known to be a marker of bone marrow recruited immune cells [34,35], we found a higher percentage of Ly6B^hi^ macrophages in DUSP3^-/-^ pulmonary cell suspensions compared to DUSP3^+/+^ control mice. The DUSP3^-/-^ macrophages as well as bone marrow monocytes are more easily recruited to LLC tumour-conditioned medium than DUSP3^+/+^ monocytes and macrophages, possibly explaining the higher number of monocyte/macrophages found in DUSP3^-/-^ LLC-bearing lungs. Ly6C^hi^ could also have been differentiated from tumour monocytes inflammatory pool, known to continuously seed tumors and renew all nonproliferating TAM subsets [24]. To investigate this hypothesis, simunltaneous staining of cells with anti-LyC6 and anti- CX3CR1 need to be included in the staining panel as these cells have been shown to be Ly6C^hi^CX3CR1^low^ [24].

Proliferation assays, using CFSE labelling, performed on BMDMs stimulated with LLC-CM showed no difference between DUSP3^+/+^ and DUSP3^-/-^ BMDMs. This result excludes the idea that the higher number of macrophages found in DUSP3^-/-^ lung homogenates could be due to higher macrophage proliferation and strengthens the involvement of cell migration. The enhanced recruitment of DUSP3^-/-^ macrophages is further confirmed by an *in vitro* migration assay. Hence, upon stimulation with LLC-conditioned medium, BMDMs and monocytes from DUSP3^-/-^ mice migrated faster than the ones from DUSP3^+/+^ mice. This was not the case when cells were stimulated with B16-conditioned medium. In addition, this difference was not observed for neutrophils. These results are in line with *in vivo* observations on B16 lung metastasis. Indeed, the percentage of macrophages was identical in B16-bearing lungs of DUSP3^+/+^ and DUSP3^-/-^ mice and the percentage of Ly6B^hi^ macrophages was equal if not lower in DUSP3^-/-^ mice compared to control mice. This further proves that there is a higher infiltration of macrophages in DUSP3^-/-^ mice after LLC i.v. injection and that they are responsible for the enhanced lung metastasis formation. In fact, although these macrophages did not influence *in vitro* LLC proliferation or migration (S4 Fig), they may be responsible for the enhanced *in vivo* LLC proliferation as judged by the significant increase of Ki67^+^ cells in DUSP3^-/-^ lungs bearing tumours compared to the control lungs (Fig 8). Several studies have indeed demonstrated that the density of TAMs positively correlates with tumor growth and is associated with a poor prognosis. The mechanisms involved are poorly understood but several studies have shown that TAMs secrete several factors, such as IL10, TGF-β, Arg1, CCL-17, CCL22 and galectin-3 that contribute to immunosupresion. Furthermore, TAMs could also activate and protect tumor stem cells, stimulate their proliferation, promote angiogenesis and metastasis [18][19][25][26].

One explanation for the higher recruitment of macrophages under LLC but not B16 influence would be that the profiles of cytokines and chemokines secreted by LLC or B16 differ. For example, tumour cells are the major source of CCL2 in Lewis lung carcinomas while B16 cells express low level of CCL2 [36]. Cytokines that are strong monocyte/macrophage chemottractants such as CSF-1, TGF-β or CCL7 may thus also be differentially expressed between these two cell lines, which may explain the differential recruitment of macrophages to the metastatic site of LLC and B16 cells. It is likely that one cytokine or the combination of some cytokines secreted by LLC and not B16 may favour the recruitment of DUSP3^-/-^ macrophages that could explain the differential recruitment of macrophages to the metastatic site of LLC versus B16 cells. Consequently, it will be interesting to further investigate the precise signalling pathway implicating DUSP3 in the enhanced LLC tumour cell dissemination to lung tissues and the exact molecular role of DUSP3 in this event.

In line with our findings, a recent study showed that in NSCLC system, DUSP3 overexpression were associated with reduced cell migration and vis versa. The authors of this study also showed that DUSP3 interacts with FAK (focal adhesion kinase) and demonstrated that the negative correlation between cell migration and DUSP3 expression was at least partially due to its inhibitory effect on FAK and to suppression of EGFR whish is involved in cell chemotaxis [37]. Such mechanisms have to be investigated in our model.

Another intriguing role of DUSP3 is its function as a pro-angiogenic factor [14]. Since angiogenesis has been regarded as essential for tumour growth and metastasis, our presented results seem contradictory to our previous observations [14]. However, studies of many human tumours suggest that tumours can grow and metastasize without angiogenesis. Indeed, several reports showed evidence for tumour resistance and adaptation to anti-angiogenic therapy leading to more metastasis [38,39]. Moreover, low doses of angiogenesis inhibitors resulted in vessel normalization, well oxygenated tumours and increased effectiveness of chemotherapy [40]. On the other hand, growing evidences indicate that tumour cells may use alternative mechanisms for blood supply. For example, vessel co-option, a procedure of hijacking the blood vessels in surrounding normal tissue, was reported in vascularized tissues such as brain, lung, and liver [41]. Vessel co-option may occur in tumours independently of sprouting angiogenesis and there is increasing evidence supporting the use of this alternative blood supply in metastasis and resistance to anti-angiogenic therapy [42,43]. Although the impact of vessel co-option in our model is still to be demonstrated, we can assume that metastasis in our tumour model is not dependent on angiogenesis.

## Conclusion

In this study, we provide evidences for an unexpected role of DUSP3 in cancer metastasis. In DUSP3-deficient mice, the enhanced LLC-lung metastasis involves a better recruitment/migration of macrophages, which in turn, favour metastasis growth. In conclusion, we show that DUSP3 acts as an anti-metastatic agent by regulating the migration monocytes/macrophages to the site of metastasis.

## Supporting information

S1 Fig Gating strategy used to identify immune cell populations in DUSP3^+/+^ and DUSP3^-/-^ LLC-bearing lungs. Cells were isolated from enzymatically digested mice lungs and, after exclusion of doublets and debris, immune cells were identified by CD45.2 and CD11b staining. A sequential gating strategy was then employed to first identify populations expressing specific markers: alveolar macrophages (alveolar MΦ) (Siglec-F^+^ Ly6G^-^), eosinophils (Siglec-Fint Ly6G^-^), neutrophils (Ly6G^+^Siglec-F^-^). Distinct populations of Ly6G^-^/Siglec-F^-^ monocytes/macrophages (Mo/MФ) were further identified based on their expression of Ly6C and MHC-II.
(EPS)

S2 Fig Ly6B cell surface expression on macrophages from B16-tumour bearing lungs. (**A**) Representative dot plot of Ly8B^+^/MHC-II^+^ populations out of CD11b^+^/Ly6G^-^/Siglec-F^-^ gated cells from DUSP3^+/+^ and DUSP3^-/-^ B16 bearing mice. (**B**) percentage of Ly6B^hi^, Ly6B^int^ and Ly6B^low^ macrophages in DUSP3^+/+^ and DUSP3^-/-^ mice. n = 5 for each genotype.
(EPS)

S3 Fig Efficiency of specific macrophage depletion using clodronate-liposomes. **(A)** Gating strategy and **(B)** percentages of M1-like and M2-like macrophages in peritoneal cavity of mice from each condition. **(C)** Gating strategy and **(D)** percentage of Ly6B^+^ cells in LLC-bearing lung cell suspension from DUSP3^+/+^ and DUSP3^-/-^ mice. PBS: Empty-liposomes; CL: clodronate liposomes.
(EPS)

S4 Fig *In vitro* proliferation of BMDMs and LLC cells and in migration of LLC cells. **(A)** LLC cells migration in presence of DUSP3^+/+^ and DUSP3^-/-^ BMDM-conditionned medium. BMDM: Bone Marrow-Derived Macrophages. **(B-D)** proliferation of LLC and BMDMs. **(B-C)** CFSE was incorporated into BMDMs and cells were cultured for 24h and 48h in presence of LLC-conditioned medium. Mean fluorescence intensity of CFSE is shown in **(B)** and quantification is shown in **(C)**. **(D)** LLC cells proliferation was measured in presence of DUSP3^+/+^ and DUSP3^-/-^ BMDM-conditioned medium by the quantification of the bioluminessence.
(EPS)

S1 File Supplemental methods. (DOCX)
