# Supplementary material for: Dusp3 deletion in mice promotes experimental lung tumour metastasis in a macrophage dependent manner
Source: PLoS One. 2017 Oct 11;12(10):e0185786. doi: 10.1371/journal.pone.0185786 (PMC5636116; doi:10.1371/journal.pone.0185786)
Supplement: S1 File — (DOCX) [file pone.0185786.s005.docx]

# Supplemental methods

*BMBM CFSE proliferation assay*

BMDMs were washed twice with pre-warmed PBS. 5μM of CellTrace CFSE (Invitrogen) were added and cells were incubated for 15 min at 37°C. Labelling was stopped by removing the medium. Cells were washed twice with pre-warmed PBS, then stimulated with 2 mL of LLC-CM for 24h and 48h. CFSE intensity was assessed by flow cytometry. Data were analysed using FlowJo software

*LLC migration assay*

LLC cells migration was assessed using 5 μm polycarbonate transwells (Corning). The membrane was equilibrated for 1h at 37°C with 600 μL and 100 μL of RPMI in the lower chamber and in the upper chamber, respectively. 1x10^5^ cells were plated in 100 μL of medium in the upper chamber while 600 μL of RPMI were added to the lower chamber. DUSP3^+/+^- or DUSP3^-/-^-BMDM-conditioned-medium was added to the lower chamber. The cells were incubated for 18h at 37°C. The cells migrated to the lower chamber were recovered and counted using the Millipore ScepterTM cell counter after gating on live cells using the Scepter Software 1.2 (Millipore). Migration index was calculated as the number of transmigrating cells in the presence of chemokine per number of cells transmigrated in the absence of the chemokine multiplied by 100.

*LLC proliferation assay*

2x10^4^ LLC-luciferase positive cells were plated in 48-well plates, washed twice with pre-warmed PBS and incubated with 2ml of RMPI, DUSP3^+/+^- or DUSP3^-/-^-BMDM-conditioned medium for 8h, 16h and 24h at 37°C. Cells were then incubated with 30mg/mL of luciferine potassium salt (Promega) for 12 minutes and the photons emitted by the luciferase activity were detected for each time point. The bioluminescence was quantified using the Living Image Software (Caliper Life Sciences).
